# Clueless with fewer cues from endothelin. Commentary: Venous endothelin guides sympathetic innervation of the developing mouse heart

**DOI:** 10.3389/fcell.2015.00054

**Published:** 2015-09-24

**Authors:** Cedric Viero

**Affiliations:** Experimental and Clinical Pharmacology and Toxicology, Medical Faculty, Saarland UniversityHomburg, Germany

**Keywords:** cardiogenesis, sympathetic nervous system, endothelin receptor, neurohumoral regulation, regeneration

The potent vasoconstrictor endothelin modulates cardiac function and has been associated with the onset of hypertrophy, heart failure, myocardial infarction, hypertension, and arrhythmia (Lehmann et al., 2014). In addition, this 21-amino-acid peptide, also referred to as neurohormone, plays a decisive role in the development of the cardiovascular system (see for instance: Arima et al., 2012). The current commentary will highlight the fine-tuned connection between developing sympathetic neurons and cardiac morphogenesis.

The development of the cardiovascular system starts at embryonic day E7.5 in the mouse, as soon as cells from the late primitive streak move to the anterior region of the embryo to form the cardiogenic mesoderm (Van Vliet et al., 2012). Likewise sympathetic innervation of the heart takes place during embryogenesis, with axons from the stellate ganglia, principally, following blood vessels to reach their target and make synapses; a phenomenon relying on the expression of nerve growth factor (NGF; Ieda et al., 2004). For the first time, the work by Manousiouthakis and collaborators exposes the clear coordination orchestrated by endothelin between the projections of the cardiac sympathetic innervation and cardiogenesis. At E14, axons begin to extend and primarily project onto the dorsal side of the heart (Manousiouthakis et al., 2014). The *in situ* hybridization experiments show that the endothelin receptor genes (A and B) are expressed in neurons of the stellate ganglia at E14.5, while at the same stage endothelins 1 and 3 were detected in the endothelium of the left and right superior vena cavae, of the sinus venosus (and of their small capillaries) and in cardiac myocytes of the epicardium. After a final growth of the axon bundles, the definitive organization of the innervation is achieved by E17.5. The communication describes that the stellate ganglia nerves follow venous vessels in a unique manner, and not arteries as expected for sympathetic axons, to reach the developing sino-atrial node and myocardium. This finding echoes and supplements the pioneering work of Mukouyama and colleagues who provided evidence that distal sympathetic axons use, during an intermediate stage of innervation, large diameter coronary veins of the subepicardial area to grow along the dorsal ventricular wall (Nam et al., 2013). The expression of neurotrophins and the secretion of NGF in particular were shown to be primarily involved in this patterning of the developing mouse heart (Hasan, 2013).

These important results from the Manousiouthakis' article are supported by experiments performed in mice deficient for the endothelin receptor A, for endothelin 1, and for the endothelin converting enzyme. Using explant assays, the investigators thus demonstrate that endothelin 1 produces the key tropic effects for the neurite outgrowth from the stellate ganglia toward the venous segments.

Beside the global knockout strategy, the authors employed conditional mutant models to address the issue of tissue specificity and discard any unrelated phenotype: (i) the *Tyrosine Hydroxylase Cre/Endothelin receptor A* mutant is sympathetic neuron-specific, (ii) the *receptor tyrosine kinase Tie2 Cre/Endothelin 1* mutation targets the vascular endothelial lineage, (iii) the *t-box transcription factor Tbx18 Cre/Endothelin 1* embryos are deficient in their venous and coronary smooth muscles. It should be mentioned that although tyrosine hydroxylase conventionally serves as a specific marker for sympathetic neurons, control immunostaining of parasympathetic axonal projections with vesicular acetylcholine transporter or choline acetyltransferase would have been appreciated. Furthermore the clear distinction between the two limbs of the autonomic nervous system does not appear to be so straightforward. Indeed subsets of sympathetic neurons can also be cholinergic (Anderson et al., 2006), especially in the embryo (Higgins et al., 1981), transient expression of noradrenergic genes was observed in parasympathetic ganglia during development (Müller and Rohrer, 2002), and cardiac sympathetic neurons may undergo a transdifferentiation into cholinergic neurons in pathophysiological conditions (Kanazawa et al., 2010). Therefore the latter aspects shall be taken into consideration for the data analysis and interpretation of the results, mainly with regard to the phenotype of the knockout mice.

Noteworthy is that the *Tyrosine Hydroxylase Cre/Endothelin receptor A* and *receptor tyrosine kinase Tie2 Cre/Endothelin 1* mutant mice developed normally at the postnatal stage, albeit with a diminished sympathetic innervation. Nevertheless their stress response was impaired, as shown by electrocardiographic measurements upon an amphetamine (inducing the release of noradrenaline) challenge. Hence a correct sympathetic wiring of the heart depending on endothelin does not seem to be essential for a proper cardiac function in resting conditions, but we can deduce that it will be critical to adapt to stressful stimuli during the course of a life.

Disrupted endothelin homeostasis occurs predominantly at the adult stage and rarely during development, and is characterized by elevated concentrations (Lehmann et al., 2014) and not by a lack of hormone. Consequently, chronic increased endothelin concentrations in patients might lead to the desensitization of its corresponding receptors. Considering the hypothesis of the re-expression of a fetal program during cardiac remodeling induced by a pathological state, we would be then in the case of a deficiency in endothelin receptor A during regenerative maturation (implying a possible sympathetic re-innervation process; see Kimura et al., 2012). In turn, due to a reduced innervation as it might be expected, such a situation would reduce the responsiveness to conditions of stress after recovery according to the study commented here.

To go further, rapid changes in blood flow and the resultant shear stress during development of the cardiovascular system can be a factor of downregulation for endothelin 1 in arteries, and most likely in veins (Morawietz et al., 2000; Longchamp et al., 2014). An abnormal shear stress could hence contribute to the perturbation of cardiac innervation as described above.

Global knockout strategies targeting endothelin 1 or the endothelin receptor A affect craniofacial formation and heart development (more specifically leading to aortic arch malformations and ventricular septal defect), distinct phenotypes also found in congenital syndromes including Pierre-Robin syndrome, DiGeorge syndrome, and velo-cardio-facial syndrome (Kurihara et al., 1995). While these syndromes are characterized by deletions in chromosome 22, the human endothelin 1 gene is associated with chromosome 6, excluding any direct genetic involvement. However endothelin 1 gene polymorphisms play a role as a modifier in other disorders such as dilated cardiomyopathy (Matsa et al., 2014).

Beside the implication of the mechanism presented in the commented article (i.e., endothelin-dependent cardiac innervation and adaptation to stress) in the context of evolution, as proposed by the authors, putative therapeutic strategies arising from these results and earlier studies are worth discussing here (Figure 1). Denervation can affect parasympathetic ganglia, as in the Chagas' disease (see Teixeira et al., 2001), but also cardiac sympathetic nerves, as in the early onset of Parkinson's disease (Orimo et al., 2007). Whilst cardiovascular pathologies involving sympathetic plasticity are mostly characterized by hyperinnervation, which may lead to the generation of arrhythmia, sympathetic denervation also occurs in myocardial infarction and heart failure (Hasan, 2013). In the latter case, following the *nerve sprouting* hypothesis, aberrant regenerative growth may result in heterogeneous myocardial areas containing a mixture of denervated and hyperinnervated patches, therefore increasing the propensity for electrical instability (Chen et al., 2007). Thus, in spite of the controversy about the clinical efficacy of endothelin receptor antagonists in heart failure in particular, the ability to manipulate the endothelin system locally and specifically to promote a successful re-innervation or support the endogenous process of sympathetic regeneration (Kimura et al., 2012) opens novel perspectives of targets with regard to treatment in the field of cardiovascular diseases, and might help to prevent fatal arrhythmia and sudden cardiac death.

**Figure 1:**
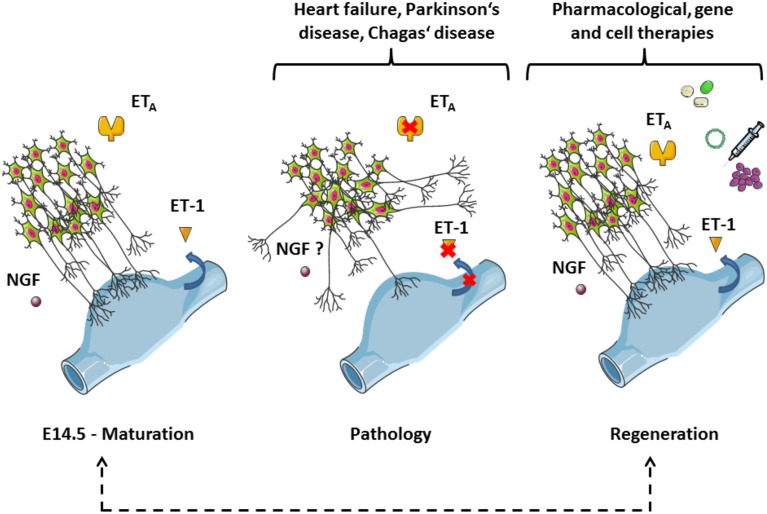
**Sympathetic axons follow veins in an endothelin-dependent manner during cardiac development: implications for disease, rejuvenation, and therapy**. The panel is based on Figure 3 of the commented article (Manousiouthakis et al., 2014). This simplified scheme depicts neurites of the stellate ganglia reaching sinus venosus segments (blue parts). The process occurs during the embryogenesis from day 14.5 (E14.5) onwards and is controlled by guidance cues from the endothelin signaling (left-hand side). Of note, NGF plays an important trophic role at this stage. Aberrant outgrowths take place when the endothelin signaling is disrupted, which might be a mechanism for denervation triggered by pathological conditions (middle). The red crosses indicate putative molecular targets of the dysfunctions (blue arrow: secretion). Whether NGF expression and concentration are also affected in this model will require further investigations. The understanding of the sympathetic innervation of the heart during development should provide useful information to stimulate endogenous regeneration as a possible treatment for cardiovascular diseases (right-hand side). NGF, Nerve Growth Factor; ET-1, endothelin 1, ET_A_, endothelin receptor type A. The figure was produced using Servier Medical Art.

## Funding

CV was supported by the Deutsche Forschungsgemeinschaft (SFB 894) and a travel grant from the Physiological Society (UK).

### Conflict of interest statement

The author declares that the research was conducted in the absence of any commercial or financial relationships that could be construed as a potential conflict of interest.
